# Do concerns about COVID-19 impair sustained attention?

**DOI:** 10.1186/s41235-021-00303-3

**Published:** 2021-05-27

**Authors:** Jihyang Jun, Yi Ni Toh, Caitlin A. Sisk, Roger W. Remington, Vanessa G. Lee

**Affiliations:** Department of Psychology, University of Minnesota, 75 East River Road, S504 Elliott Hall, Minneapolis, MN 55455 USA

**Keywords:** Sustained attention, COVID-19, Vigilance decrement, Mental health

## Abstract

The novel coronavirus disease 2019 (COVID-19) has considerably heightened health and financial concerns for many individuals. Similar concerns, such as those associated with poverty, impair performance on cognitive control tasks. If ongoing concerns about COVID-19 substantially increase the tendency to mind wander in tasks requiring sustained attention, these worries could degrade performance on a wide range of tasks, leading, for example, to increased traffic accidents, diminished educational achievement, and lower workplace productivity. In two pre-registered experiments, we investigated the degree to which young adults’ concerns about COVID-19 correlated with their ability to sustain attention. Experiment 1 tested mainly European participants during an early phase of the pandemic. After completing a survey probing COVID-related concerns, participants engaged in a continuous performance task (CPT) over two, 4-min blocks, during which they responded to city scenes that occurred 90% of the time and withheld responses to mountain scenes that occurred 10% of the time. Despite large and stable individual differences, performance on the scene CPT did not significantly correlate with the severity of COVID-related concerns obtained from the survey. Experiment 2 tested US participants during a later phase of the pandemic. Once again, CPT performance did not significantly correlate with COVID concerns expressed in a pre-task survey. However, participants who had more task-unrelated thoughts performed more poorly on the CPT. These findings suggest that although COVID-19 increased anxiety in a broad swath of society, young adults are able to hold these concerns in a latent format, minimizing their impact on performance in a demanding sustained attention task.

**Supplementary Information:**

The online version contains supplementary material available at 10.1186/s41235-021-00303-3.

## Introduction

The widespread infections and devastating economic damage caused by the novel coronavirus disease 2019 (COVID-19) has increased stress, even in individuals who are otherwise healthy. While psychologists have begun to address these mental health challenges (Rajkumar, 2020), less understood is how concerns surrounding COVID-19 affect cognitive functions, including those important for driving, learning, and workplace productivity. In two pre-registered experiments on young adults, we explored the connection between COVID-related concerns and one important cognitive function: sustained attention.

Increasing evidence has shown that internal states, including ongoing worries, interfere with attention. Mind wandering, or engagement in task-unrelated thoughts, is already rampant, occurring 50% of the time during daily activities (Killingsworth & Gilbert, 2010). When occasional thought probes are used to assess the focus of one’s thoughts during a continuous task, participants often report task-unrelated thoughts that are grounded in their concerns (McVay & Kane, 2010; Poerio et al., 2013; Smallwood & Schooler, 2015). These findings suggest that as concerns about COVID-19 increase, people may become more distractable. However, many factors contribute to mind wandering, including task difficulty and working memory capacity (McVay & Kane, 2009; Seli et al., 2016; Smallwood & Schooler, 2015). It is unclear whether people with greater COVID-related concerns are more prone to mind wandering, and if so, whether those concerns impair sustained attention.

Other studies have found that emotion, such as fear, modulate attention. Compared with neutral stimuli, fearful stimuli tend to capture attention (Ohman & Mineka, 2001). Fear may also narrow the focus of attention to fear-inducing stimuli (Steblay, 1992). These findings suggest that one should apply resource competition theories to the context of COVID-19 concerns. For instance, the dual competition framework considers task-irrelevant threats to be sources of distraction that consume attentional resources that could otherwise be devoted to ongoing tasks (Pessoa, 2008).

However, COVID-related concerns may give rise to general anxiety, rather than specific fears. The relationship between anxiety and attention is less straightforward than that between fear and attention (Robinson, Krimsky, et al., 2013; Robinson, Vytal, et al., 2013). For example, although patients with anxiety disorders may have deficits in brain regions regulating cognitive control, they do not always perform more poorly on cognitive control tasks (Eysenck et al., 2007; Lagarde et al., 2010). Experimentally induced anxiety, such as the threat of receiving an electric shock in an experiment, is associated with worse cognitive control in some tasks but not others (Choi et al., 2012; Robinson, Krimsky, et al., 2013; Robinson, Vytal, et al., 2013). These conflicting findings raise questions about the degree to which internal states, such as COVID-related worries, affect attention.

Other evidence for a connection between severe concerns and cognitive performance comes from studies on the effects of poverty. Mani et al. (2013) showed that poverty impairs cognitive control. In one study, they asked shoppers at a New Jersey shopping mall to think about a car repair that some were told would cost $1,500 and others were told would cost $150. Participants were categorized as poor or rich based on their income level. They completed the Raven’s Progressive Matrices and a spatial compatibility task. Performance was comparable between the poor and rich participants when contemplating the affordable car repair, but performance was severely impaired when the poor (but not the rich) participants contemplated the expensive care repair. In another experiment, seasonal sugarcane farmers from India were tested in a numerical Stroop task either before harvest, when the farmers were poorer, or after harvest. Performance was significantly worse before than after harvest, an effect that could not be explained by differences in nutrition intake or physiological measures of stress. Mani et al. (2013) proposed that concerns derived from poverty exert a cognitive load, depleting the available resources that poor individuals have in performing cognitive tasks (Shah et al., 2012). The negative effects of scarcity are not restricted to poverty. They also affect people who are scarce in other resources, such as time (Cannon et al., 2019). These findings suggest that as access to financial or healthcare resources declines during the COVID-19 pandemic, performance on attention tasks may also suffer.

Together, studies on mind wandering, emotion, and the economics of scarcity hint at the possibility that rising concerns about COVID-19 may interfere with attention. However, conflicting evidence on the relationship between anxiety and attention raises questions about whether moderate increases in concerns about one’s health and financial well-being yield measurable effects. This is an important question because in a pandemic like COVID-19, current concerns are elevated across a broad swath of society. If even moderate concerns impair performance to a measurable degree, this may warrant changes to safety and workplace practice. Conversely, if attentional functions are robust in the presence of moderate concerns, this allows for a shift of focus from the broad but moderate anxiety to narrower but more severe mental health issues.

To investigate the connection between COVID worries and attention, we administered a continuous performance task (CPT) that produces robust individual differences. This task was adopted from Esterman et al. (2013), which presented participants with a continuous stream of natural scenes for several minutes. Participants pressed one button in response to city scenes that occurred 90% of the time and withheld responses to mountain scenes that occurred 10% of the time. Using a variant of this task, Rosenberg et al. (2016) tested young adults while they underwent functional magnetic resonance imaging (fMRI). Participants showed reliable individual differences in their CPT performance. In addition, a brain connectivity network built on these participants’ fMRI data successfully predicted the severity of ADHD symptoms in children, providing strong evidence for its external validity. Other studies showed that the scene CPT was highly demanding, producing rapid performance decline after just two minutes on the task (Esterman et al., 2013; Jun et al., 2019). Although Esterman et al. (2013)’s task used images with gradual onset, key features of the task were replicated when images were presented with an abrupt onset (Jun & Lee, submitted). These findings make the scene CPT an ideal choice for assessing the impact of COVID-19 on attention.

Here, we used a correlational approach to explore the relationship between pandemic-related concerns and sustained attention. We recruited young adults from a behavioral research crowdsourcing site (Prolific.co). Experiment 1 was conducted in June 2020, during an earlier phase of the pandemic. Most participants resided in European countries that were experiencing increasing severity of the pandemic. Experiment 2 was conducted in February 2021 on participants from the US. This corresponded to a later phase of the pandemic. Vaccination had begun for older adults in the US but was not yet available for young adults. As conceptual replications of each other, the two experiments allowed us to assess the generality of the findings at different time points of the pandemic and in different geographic locations.

## Experiment 1

Participants in Experiment 1 first completed a survey that assessed health- and finance-related concerns in light of the pandemic. They then completed the scene CPT, comprising two, 4-min blocks, during which participants viewed a continuous stream of scenes and made button-press responses to frequent city scenes while withholding responses from infrequent mountain scenes. If COVID-related concerns significantly reduce attentional resources available for the CPT, then participants expressing higher concerns in the pre-task survey should perform more poorly on the CPT and show a larger and faster performance decline over time. Conversely, if participants are able to exert control over their concerns about COVID-19 and minimize their negative impact, then COVID-related concerns may not significantly correlate with performance on the CPT.

### Method

#### Pre-registration

This study was pre-registered on the Open Science Framework (https://osf.io/ay8pq/). The pre-registration included details about the study purpose, hypotheses, sample size determination, design, and analysis plan.

#### Participants

The final dataset came from 161^1^ participants, including 103 males and 58 females, with a mean age of 23 years (range 18–44; SD = 5.2). Participants were recruited from Prolific.co, an online website for behavioral research. They met the inclusion criteria: 18–45 years of age; fluent English; normal vision; and no history of neurological or psychiatric conditions. Participants came from 21 countries throughout Europe (87%) and the Americas (12%; 2% from the USA). They provided informed consent through Qualtrics and received $2 compensation. The study was approved by the University of Minnesota’s Institutional Review Board. Additional demographic information can be found in Additional file 1 (Table S1).

##### Sample size determination

Sample size, determined using G*Power (Faul et al., 2007), was designed to detect a moderate effect size of 0.3 in four planned correlation analyses (2 sustained attention indices × 2 types of concerns). This effect size was based on Rosenberg et al. (2013)’s finding of a significant correlation between error rates in the scene CPT and attentional lapses in daily life (*r* = 0.47). A sample of 161 achieved a power of 0.95 in detecting an effect size of 0.3 at a Bonferroni-corrected alpha of 0.0125.

##### Data exclusion

Data from 28 additional participants were excluded according to pre-registered data exclusion criteria. Four participants were excluded for quitting the study before starting the CPT. Five participants were excluded for responding less than 30% of the time in the CPT. Two others failed to choose “2” on the survey when asked to. Sixteen participants were excluded for failing to provide consistent responses to two identical but reversely-worded items, rated on a 7-point scale (the two items were “I am worried about the coronavirus” and “I am not concerned about the coronavirus”). Responses were considered consistent if the sum of the two ratings was between 6 and 10. Finally, one participant was excluded for performing below 4 standard deviations of the group mean in the scene CPT.

#### Procedure

Participants first completed a COVID-19 survey through Qualtrics. This was followed by the scene CPT administered on Pavlovia.org on the participants’ own computers.

##### COVID-19 survey

We developed the survey based on existing COVID-19 surveys (Conway et al., 2020; Grasso et al., 2020) and the PhenX toolkit (phenxtoolkit.org/covid19). It began with demographic questions, followed by questions about COVID-19’s infection history, current concerns, and compliance with public health recommendations. The survey can be found in Additional file 1: S1.

The items assessing current concerns included two health-related items, two finance-related items, two items assessing anxiety around crowds, and two items assessing general concerns. The last two were reversely-worded to index response consistency (i.e., “*I am worried…”* versus “*I am not concerned…*”). Responses were recorded on a 7-point scale, ranging from “not true of me at all” (1) to “very true of me” (7). There was also an attention check asking participants to respond “2.” Table 1 lists the relevant items assessing current concerns.

**Table 1:** Survey items used in Experiment 1 to assess current concerns. Ratings were obtained on a 7-point scale

Item category	Item content
Health-related concerns	*I am worried that I or people I love will get sick from the coronavirus*
	*I am worried that the coronavirus will delay the treatment of other illnesses that I or people I love may have*
Finance-related concerns	*I have lost job-related income due to the coronavirus*
	*I am concerned about my job security due to the coronavirus*
Anxiety around crowds	*I am stressed around people outside of my household because I worry I’ll catch the coronavirus*
	*I have tried hard to avoid people outside of my household because I don’t want to get sick*
General concerns: reversely worded to index response consistency^a^	*I am worried about the coronavirus*
	*I am not concerned about the coronavirus*
Attention check	*Please choose “2”*

^a^These items were included to check for response consistency. Responses were considered consistent if the sum of the responses to these two items was between 6 and 10

Additional items assessed compliance with public health recommendations, such as hand washing and social distancing behaviors.

##### Scene CPT

In the scene CPT, participants first viewed a set of 10 city images and pressed “c” after each one. They were then shown 10 mountain images for 1 s each and asked not to respond. The scenes were grayscale and circular (radius = 128 pixels). After this familiarization phase, participants practiced the task. The scene CPT was modeled after Esterman et al. (2013), which presented participants with a continuous stream of scenes at a pace of 800 ms/scene. Participants were asked to press “c” in response to cities and withhold response to mountains. The ratio of city to mountain trials was 9:1. Unlike Esterman et al. (2013) which used images that onset gradually, in our study the scenes were presented one at a time in clear view for 560 ms, followed by a 240 ms blank. The clear view version was used because the precise timing of the gradual onset was difficult to achieve in online testing. A recent study showed that performance on the gradual version was strongly correlated with that on the clear-view version of the scene CPT (*r* = 0.68; Jun & Lee, submitted).

To ensure that participants understood the task, feedback was provided during practice, reminding participants to press “c” if they missed a city, or to withhold response if they responded to a mountain. Practice ended after 30 correct responses. The maximum number of practice trials any participant needed was 38.

**Fig. 1:**
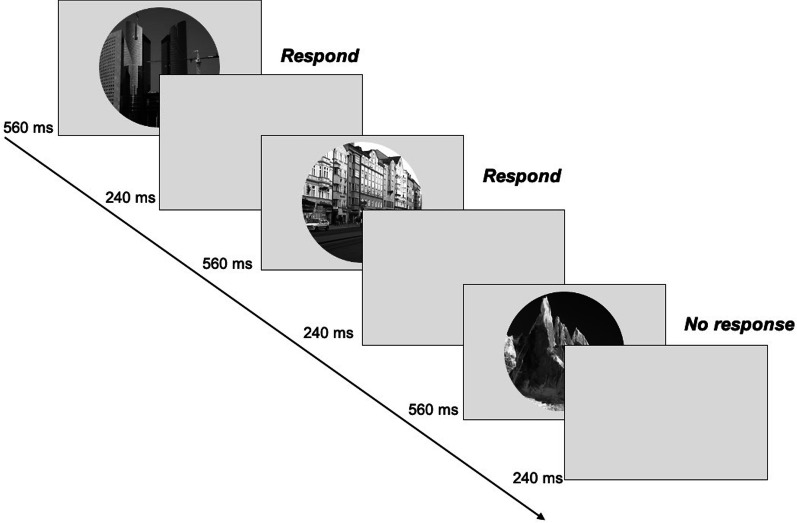
The scene continuous performance task (CPT)

Following practice, participants completed the scene CPT without feedback for two, 4-min blocks, with a minimum 10 s break between blocks (Fig. 1). The duration of the scene CPT was within the typical duration of CPT variants (e.g., 4-min in Robertson et al., 1997; 12-min in Rosenberg et al., 2016). Presentation pace was identical to that used in practice. The entire 8-min CPT included 600 trials, divided into four time bins of 150 trials each. For each time bin, the sequence of 150 images was randomly composed using the set of 10 cities and 10 mountains, with the constraints that (i) cities comprised 90% of the trials, (ii) a specific image did not occur consecutively, and (iii) the longest run of cities (without a mountain) did not exceed 25. The 150-trial sequence differed for the four time bins. To control for stimulus differences, all participants were tested using the same four 150-trial sequences. However, the order of the four sequences was counterbalanced to ensure that differences between blocks or across time bins could not be attributed to stimulus differences. Participants were randomly assigned to four possible orders for counterbalancing.

After completing the scene CPT, participants clicked on a continuous response scale (0–50%) to estimate the proportion of trials that contained mountains.

#### Data analysis

We followed the pre-registered analysis plan.

For the COVID-19 survey, we tested the consistency between the two health-related items, and between the two finance-related items. We performed a factor analysis on the eight items assessing COVID-related concerns. Exploratory analysis examined the association between concerns and demographics.

For the scene CPT, raw data were transformed to correct for slow responses made on trial N that got registered on trial N + 1. The transformation followed published procedures (Esterman et al., 2013) and was pre-registered (Additional file 1: S4). For example, a trial with an RT of 20 ms recorded on trial N + 1 was corrected as a response made at 820 ms on trial N. This correction affected 1.27% of trials with a response. Following data transformation, we computed omission errors (failure to respond to cities) and commission errors (erroneous responses to mountains). Following previous studies (Helton & Russell, 2011; Jun et al., 2019), we computed *A’* (Grier, 1971; Stainslaw & Todorv, 1999) as a measure of detection sensitivity. Objections can be raised to the use of *A’* (Verde et al., 2006)*,* so in Additional file 1: S5, we report *d’* results. In our study, *d’* was strongly correlated with *A’* (Pearson’s *r* = 0.96 in Experiment 1 and *r* = 0.95 in Experiment 2). The two measures yielded the same pattern of results.

There were four planned correlations, produced by crossing two types of concerns (health and financial concerns) with two indices of sustained attention (mean *A’* and the reduction in *A’* across blocks). Exploratory correlations included additional measures of concerns, such as those derived from the factor analysis.

### Results and discussion

1. COVID-19 survey

We measured Cronbach’s alpha between the two items assessing health-related concerns, and between the two items assessing finance-related concerns. The measured alpha—0.57 for health and 0.63 for finance—were below the cutoff of 0.70 for internal consistency. Thus, health and financial concerns are multifaceted, justifying the inclusion of two items in each category to capture concerns.

To understand the underlying structure of the 8 items assessing COVID-related concerns, we conducted an exploratory, principal axis factor analysis with varimax rotation. Two factors were extracted based on eigenvalues higher than 1 and inspection of scree plots. Table 2 displays the items and factor loading for the rotated factors. Loadings lower than 0.3 were disregarded to improve clarity. This analysis revealed two factors. After rotation, Factor 1 explained 40.6% of the variance, with factor loadings from 0.35 to 0.92. It included both health items, both crowd items, and both items on general concerns. Factor 2 explained 12.5% of the variance after rotation, with factor loadings from 0.63 to 0.73. It included both finance-related items. The factor analysis supported our pre-registered plan of separately analyzing health and financial concerns.

**Table 2:** Principal component factor analysis for COVID-19 concern questionnaire (N = 161)

Items	Factor loading	
	1	2
1. I am worried about the coronavirus	.92	
2. I am not concerned about the coronavirus	.83	
3. I am worried that I or people I love will get sick from the coronavirus	.79	
4. I am stressed around people outside of my household because I worry I'll catch the coronavirus	.72	
5. I have tried hard to avoid people outside of my household because I don't want to get sick	.65	
6. I am worried that the coronavirus will delay the treatment of other illnesses that I or people I love may have	.35	
7. I am concerned about my job security due to the coronavirus		.73
8. I have lost job-related income due to the coronavirus		.63
*Eigenvalues*	3.25	1.00
*% of variance*	40.62	12.46

Additional file 1: S3 summarizes results from additional, exploratory analyses on the correlations among COVID-19 survey items.

2. Scene CPT

**Fig. 2:**
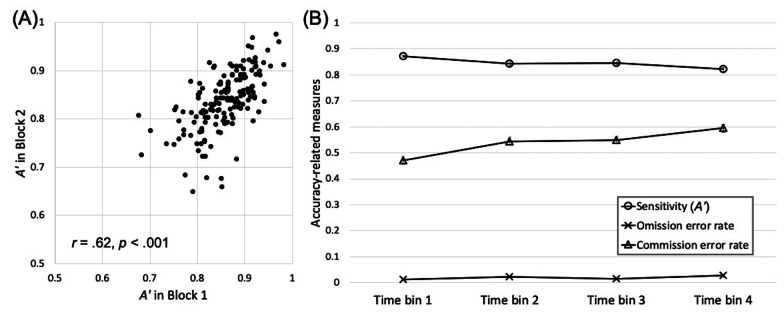
Results from the scene CPT of Experiment 1: **a** Scatterplot illustrating the correlation in A’ between block 1 and block 2; **b** Changes in A’ and error rates across the four 2-min-long time bins. Error bars show ± 1 S.E. of the mean. Some error bars may be too small to see

We replicated key characteristics of the scene CPT in the geographically diverse sample. First, demonstrating stable individual differences, the correlation coefficient for *A’* between the two CPT blocks was significant, Pearson’s *r* = 0.62, *p* < 0.001 (Fig. 2, Left).

Second, participants’ CPT performance showed rapid decline over time (Fig. 2, Right). In *A’*, an ANOVA using block (first vs. second) and time bin (first vs. second time bin of each block) as within-subject factors showed that *A’* declined both between blocks, *F*(1, 160) = 29.11, *p* < 0.001, *η*_*p*_^2^ = 0.15, and within a block, *F*(1, 160) = 48.74, *p* < 0.001, *η*_*p*_^2^ = 0.23*,* with no significant interaction, *F*(1, 160) = 0.28, *p* = 0.60, *η*_*p*_^2^ < 0.01*.* As shown in Fig. 2, the decline in *A’* originated primarily from an increase in commission errors (i.e., failure to withhold response to mountains) over time. Omission errors (i.e., failure to respond to cities) were low across all time bins.

Previous studies have linked commission errors to a failure to suppress frequent responses (Jun et al., 2019; Wilson et al., 2016). Consistent with this proposal, an analysis on response time (Additional file 1: S6) showed that responses became faster as the experiment progressed. Despite the high commission error rates, participants accurately estimated the frequency of mountains. The median estimate of the proportion of mountains was 10% (mean 12.7%), consistent with the actual proportion. The accurate estimates suggest that the high rates of falsely responding to mountains did not occur because participants mistakenly perceived more mountains than were actually present. Rather, they occurred due to response error. When we computed each participant’s frequency estimation error as the absolute deviation from 10%, we did not find a significant correlation between frequency estimation error and CPT *A’*, Pearson’s *r* = − 0.09, *p* = 0.23. Similar results were found in Experiment 2 (*r* = − 0.06, *p* = 0.38). Thus, errors on the CPT likely reflected premature responses, rather than perceptual failures.

3. Correlation between COVID-related concerns and CPT performance

**Fig. 3:**
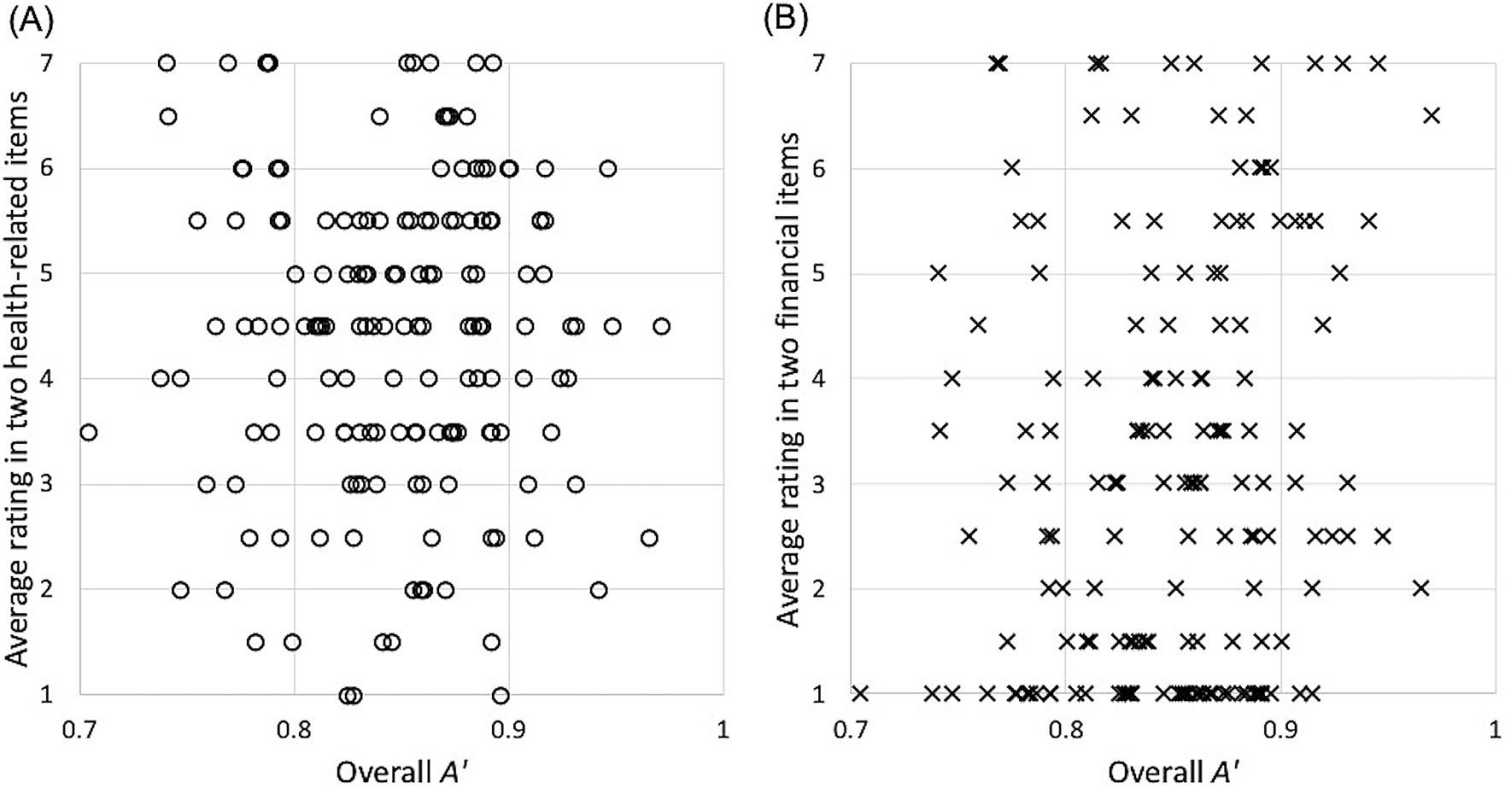
Scatterplots illustrating the lack of correlation between A′ in the CPT and pre-task COVID-related concerns: **a** A′ and health-related concerns **b** A′ and financial concerns

We conducted four planned correlation analyses (Bonferroni-corrected alpha was 0.0125) to examine the correlation between COVID-related concerns and CPT performance. Health concerns were the average rating of the two health-related items. Financial concerns were the average rating of the two finance-related items. Despite the wide range of concerns across participants and the large individual differences in CPT performance, we did not find any systematic relationship between the two. People with severe COVID-related concerns performed just as well as people who were not concerned. *A’* did not significantly correlate with either health concerns, *r* = − 0.01, *p* = 0.95, or financial concerns, *r* = 0.15, *p* = 0.052 (Fig. 3). In addition, the decline in *A’* from block 1 to block 2 did not correlate with health concerns, *r* = 0.01, *p* = 0.91, or financial concerns, *r* = − 0.13, *p* = 0.11. These results held after controlling for age, education level, income, and political orientation.

To quantify the evidence in favor of the null hypothesis, we applied a Bayesian correlation using the JASP software project with default priors (Version 13, JASP Team, 2020). In the case of a null effect, the Bayesian analysis tests whether a lack of an effect is more plausible than the presence of an effect. The Bayes Factor for the null hypothesis (BF_01_) was BF_01_ = 9.66 for the association between overall *A’* and health concerns, implying that a model omitting health concerns as a term in the *A’* analysis was 9.66 times more plausible than a model including it. The BF_01_ was 29.19 for the association between overall *A’* and financial concerns. It was 9.26 for the association between the decline in *A’* from block 1 to block 2 and health concerns, and 25.36 for the association between the *A’* decline and the financial concerns. All of the BF_01_ values present evidence against a correlation between *A’* and level of health and financial concerns (Wetzels & Wagenmakers, 2012).

To ensure that we did not miss any effects, we performed exploratory analyses using other indices of current concerns. These include (i) the average of all eight items on current concerns, and (ii) the average of the six items loading on Factor 1 of the factor analysis. Neither of these correlated with *A’* or with the reduction in *A’* over time, all *p*s > 0.09 (Table 2). We also computed the “skill index” as No-go (mountain) accuracy divided by Go (city) RT, an index previously shown to correlate with mind wandering (Seli, 2016). Skill index did not correlate with any measure of current concerns at an adjusted *p-*value of 0.0125, largest *r* = 0.17, *p* = 0.03 (Table 3).

**Table 3:** Pearson’s correlation coefficient between COVID-19 responses and CPT performance in Experiment 1. Skill index is calculated as No-go accuracy divided by Go RT

Variables	Overall *A’*	Reduction in A’ across blocks	Skill index	No-go (mountain) accuracy	Go (city) RT
*Demographic ratings*					
Age	***.19***	− .04	***.20***	***.21***	.12
Education	***.19***	− .03	***.17***	***.18***	.15
Income	.12	.06	.10	.09	.02
Political orientation	***.17***	− .10	.15	***.18***	.15
*Concerns related to COVID-19*					
Health concern	− .01	.01	.03	.04	.05
Financial concern	.15	− .13	***.17***	***.21***	***.17***
Non-financial concern (Factor 1)	.08	.05	.08	.10	.12
Average of all eight items	.13	− .01	.14	***.17***	***.17***
*Behavioral compliance*					
Social distancing	− .13	− .15	***− .16***	− .14	− .03
Hygienic behavior	− .08	− .11	− .11	− .11	− .06

In the correlation analysis between income and other variables, participants who chose the answer “I prefer not to answer this question” were excluded, leading to *N* = 142. All other analyses had *N* = 161. None of the results reached Bonferroni adjusted alpha of .001. These results held after controlling for age, education level, income, and political orientation. Boldface italics: *p* < .05 (uncorrected for multiple comparisons)

Young adults tested in Experiment 1 showed large and stable individual differences in their performance on the scene CPT. They also expressed highly divergent levels of health concerns and financial concerns related to COVID-19. However, participants with greater COVID-related concerns did not perform significantly more poorly on the scene CPT. They also did not show a significantly greater extent of vigilance decline. The correlation coefficients in the four planned correlation analyses ranged from 0.01 to 0.15. The largest of the four, a positive correlation between financial concerns and CPT performance, did not reach statistical significance. In addition, the direction of the correlation contradicted the predictions of resource competition theories. These theories predict that greater current concerns should reduce attentional resources, yielding lower *A’* (i.e., a negative correlation between concerns and *A’*). Thus, there was no evidence that pre-task COVID-related concerns interfered with sustained attention. One limitation of Experiment 1 though is the lack of a direct measurement for mind wandering during the CPT task. It is unclear whether participants who expressed higher pre-task COVID concerns carried more task-unrelated thoughts during the CPT, and if so, whether these thoughts were driven by their concerns about COVID-19. We addressed this question in Experiment 2.

## Experiment 2

Experiment 1 showed that concerns about COVID-19 did not significantly influence young adults’ performance on a continuous performance task. However, this finding was obtained during the early months of the pandemic. Most participants resided in Europe, which at the time was experiencing a milder wave of infection compared with the US. To examine the generality of the finding, in Experiment 2 we conducted a conceptual replication at a different time point and in a geographically different sample. We also increased the sample size to detect a smaller *r*, and added thought probes to examine task-unrelated thoughts during the CPT.

To this end, we recruited 204 participants from the US in February 2021. This was toward the later stage of the pandemic, with declining but still high infection rates in the US. Vaccination of vulnerable populations was underway, but not yet accessible to young adults. The sample size achieved sufficient power to detect an* r* of 0.20, at a Bonferroni-corrected alpha level of 0.0125.

An important addition to Experiment 2 was the inclusion of questions that probed participants’ frequency of task-unrelated thoughts during the CPT. The continuous nature of the CPT did not lend itself to frequent thought probes. However, it was possible to obtain self-reported estimates of task-unrelated thoughts (TUT) at the completion of the CPT. To this end, immediately after each CPT block, participants were asked to estimate the proportion of the time during the preceding task period that they had engaged in (i) any type of task-unrelated thoughts, and (ii) COVID-specific task-unrelated thoughts.

The inclusion of the TUT probes allowed us to distinguish two types of concerns: latent concerns and active concerns. The pre-task COVID survey is a measure of pre-task latent concerns. These concerns may spontaneously become active and intrude into ongoing tasks. Or they may remain in a dormant format during task performance. In contrast, the TUTs are an index of active concerns during the task. They may be more strongly related to CPT performance than are the pre-task COVID concerns.

### Method

Experiment 2 was pre-registered on the Open Science Framework (https://osf.io/ay8pq/) following the completion of Experiment 1. The two experiments were similar except for differences noted below.

#### Participants

Sample size was determined using G*Power to detect a small effect size of 0.2 in four planned correlation analyses with a power > 0.80. The final dataset came from 204 participants, achieving a power of 0.82. There were 82 males, 121 females, and 1 participant of nonbinary gender with a mean age of 29 years (range 18–45; SD = 7.5). Additional demographic information can be found in Additional file 1 (Table S1).

Participants were recruited from Prolific.co, excluding those who took part in Experiment 1. In addition to the inclusion criteria used in Experiment 1, we restricted the sample to US residents.

##### Data exclusion

Data from 76 additional participants were excluded according to the pre-registered data exclusion criteria. Specifically, twelve participants were excluded for quitting the study before starting the CPT. Two participants were excluded due to self-reported computer failure or because they had participated in a similar experiment conducted by our lab. Seven participants were excluded for responding less than 30% of the time in the CPT. Fifty-three participants were excluded for failing to provide consistent responses to the two reversely-worded survey items (i.e., “I am worried about the coronavirus” and “I am not concerned about the coronavirus”). Two participants were excluded for performing more than 4 standard deviations below the group mean on the scene CPT.

#### Procedure

Similar to Experiment 1, participants first completed a COVID-19 survey and then the scene CPT.

##### COVID-19 survey

The survey was similar to the one used in Experiment 1, except for the following changes. First, to increase the clarity of the 7-point rating scale, the scales were labeled as “I am not concerned at all about this possibility” for 1 and “I am extremely concerned about this possibility” for 7. Second, we added an assessment of overall health status and financial wellbeing. Specifically, participants were asked to rate their overall health (3-point scale: 1-good; 3-poor). In addition, they were asked to rate the difficulty of paying for the basics like food and medicine (4-point scale: 1-very hard; 4-not very hard) and to rate their household financial situation (4-point scale: 1-comfortable with extra; 4-cannot make ends meet). The first financial status item was reversely coded and the average of the two reflected financial wellbeing.

##### Scene CPT

The CPT was the same as described in Experiment 1, except that we added thought probes at three time points: after practice, at the end of block 1, and at the end of block 2. The thought probes appeared as two successive questions. First, participants were asked to indicate their overall task-unrelated thoughts (TUT) during the preceding task period. The question after each block stated: “Think about the last 4 min you spent doing the scene task. For what percentage of that time were you thinking about something unrelated to the task?” Participants responded by clicking a slider that ranged from 0–100%. Next, participants were asked to indicate their COVID-specific TUT during the preceding task period. The question stated: “For what percentage of that time were you thinking specifically about COVID-19?” Participants responded on a slider that ranged from 0–100%. The mean TUTs after each of the two task blocks were used as a measure of active concerns.

#### Data analysis

The pre-registered analysis plan was similar to that of Experiment 1, with the following differences. First, following the pre-registered data analysis plan, the CPT data analysis was conducted on the raw data without any transformation. The simplification was justified because the transformation did not affect the vast majority of responses in Experiment 1, yielding nearly identical results from the two analysis procedures. The simpler data analysis was preferred because it was easier to adopt for future replication studies. Second, the addition of the TUT changed the planned analyses. The four planned correlations involved the correlation between CPT *A’* and (1) health-related concern level, (2) financial concern level, (3) general TUT, and (4) COVID-specific TUT. The first two concern measures were obtained from the pre-task survey and can be considered as indices of latent concerns. The two TUT measures reflected active concerns during the CPT.

## Results and Discussion

**Fig. 4:**
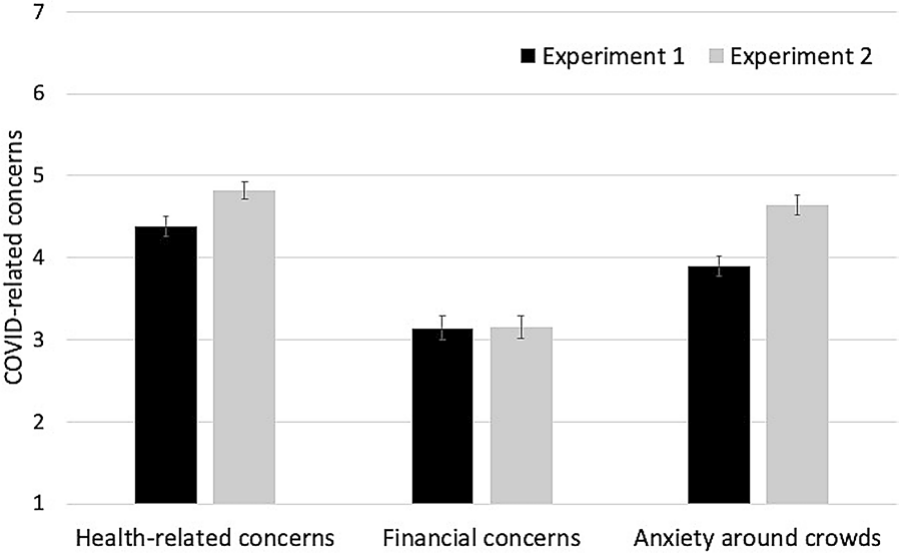
Mean rating of the COVID-related concerns on a 7-point scale (1: not concerned; 7: extremely concerned). Error bars show ± 1 S.E. of the mean

**Fig. 5:**
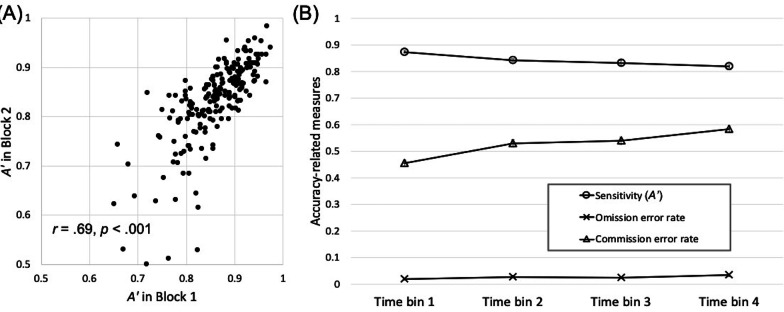
Results from the scene CPT of Experiment 2: **a** Scatterplot illustrating the correlation in A′ between block 1 and block 2 (an outlier with an A′ less than 0.5 in one of the blocks went off the chart but was included in the analysis); **b** Changes in A′ and error rates across the four 2-min-long time bins. Error bars show ± 1 S.E. of the mean. Some error bars may be too small to see

1. COVID-19 survey
  On the two new items assessing health and financial wellbeing, participants in Experiment 2 reported overall good health (mean rating = 1.25 on a 3-point scale). Their financial status rating of 1.76 on average can be described as having “enough but no extra”.
  On the items measuring COVID-related concerns, compared with those in Experiment 1, participants in Experiment 2 showed higher health-related concerns, *t*(363) = 2.83, *p* < 0.005, Cohen’s *d* = 0.30, and greater anxiety around crowds, *t*(363) = 4.27, *p* < 0.001, Cohen’s *d* = 0.45, but similar financial concerns, *t*(363) = 0.04, *p* = 0.97, Cohen’s *d* = 0.004.
  Figure 4 shows the mean COVID-related concerns across the two experiments. As a group, participants in both experiments expressed moderate health and financial concerns.
  Additional exploratory analyses on the correlations among COVID-19 survey items can be found in Additional file 1: S3.
2. Scene CPT
  Replicating Experiment 1, participants showed large and stable individual differences in the scene CPT. The correlation coefficient for *A’* between the two CPT blocks was Pearson’s *r* = 0.69, *p* < 0.001 (Fig. 5, left). A plot of the *A’* and error rates across the four time bins showed remarkably similar results between the two experiments (Fig. 5, right). As in Experiment 1, *A’* significantly declined from block 1 to block 2, *F*(1, 203) = 42.93, *p* < 0.001, *η*_*p*_^2^ = 0.18, and from the first to the second time bin of each block, *F*(1, 203) = 30.82, *p* < 0.001, *η*_*p*_^2^ = 0.13. This decline originated primarily from an increase in commission errors over time and was accompanied by increasing response speed over time (Additional file 1: S6).
3. Task-unrelated thoughts during the CPT
  On average, participants reported that their mind had wandered off the task 24.9% of the time (median = 19.0%; range 0–94%; SD = 23.3%) during the CPT. The rate of TUT specific to COVID-related thoughts was 6.5% (median = 1.1%; range 0–72%; SD = 11.9%). Both measures yielded a distribution that was extremely skewed, with a large proportion of participants providing estimates near zero. We therefore used Spearman’s *rho* rather than Pearson’s *r* when evaluating correlations.
  First, the TUTs showed significant and stable individual differences. The correlation between block 1 and block 2 was Spearman’s *rho* = 0.72, *p* < 0.001 for general TUT, and *rho* = 0.64, *p* < 0.001 for COVID-specific TUT. In addition, people who reported greater general TUT also had greater COVID-specific TUT, *rho* = 0.47, *p* < 0.001.
  Second, we examined the relationship between active concerns and latent concerns about COVID. Consistent with the idea that latent concerns may be spontaneously activated, we found that the COVID-specific TUT correlated significantly with the latent concerns about COVID-19 obtained in the pre-task survey (i.e., the average of the 8 items assessing current concerns), Spearman’s *rho* = 0.22, *p* = 0.002. Note that although the correlation was statistically significant, it was moderate in size.
4. Correlation between CPT *A’* and COVID-related concerns
  4.1. Pre-task survey of COVID-related concerns
    Replicating Experiment 1, our planned correlation analyses showed a lack of significant correlation between the CPT *A’* and either the (latent) health-related concerns, *r* = 0.012, *p* = 0.87, or the (latent) finance-related concerns, *r* = 0.014, *p* = 0.85. Re-calculating the correlations in terms of Spearman’s *rho* did not change the pattern of results: *rho* = − 0.03, *p* = 0.72 for health-related concerns, and *rho* = 0.03, *p* = 0.72 for finance-related concerns.
    To evaluate the strength of the data in relation to the null hypothesis, we conducted a Bayesian correlation analysis with default priors. The Bayes Factor for the null hypothesis (BF_01_) was BF_01_ = 12.97 for the association between *A’* and health concerns, and 13.24 for the association between *A’* and financial concerns. Thus, the data are more than 12 times as likely to occur under the null hypothesis than the alternative hypothesis of a correlation between *A’* and the pre-task level of health and financial concerns.
    As in Experiment 1, we performed exploratory analyses using other indices of current concerns. These include the average of all eight items on current concerns and the skill index (No-go-accuracy divided by Go RT). Neither of these correlated with *A’* (Table 4).
  4.2. Task-unrelated thoughts
    Task-unrelated thoughts, on the other hand, were more strongly related to performance on the CPT. First, general TUT correlated negatively with CPT *A’*, Spearman’s *rho* = − 0.174, *p* = 0.0126. Individuals reporting greater TUTs performed more poorly on the CPT. A similar trend was observed between the COVID-specific TUT and CPT *A’*, *rho* = − 0.134, *p* = 0.057. The Bonferroni-corrected alpha level was 0.0125, meaning that the correlation of CPT *A’* with general TUT, but not with COVID-specific TUT, approached significance.
  4.3. Data exclusion
    In Experiment 2, we excluded 53 participants who failed to provide consistent responses to the two reversely worded survey items (i.e., “I am worried about the coronavirus” and “I am not concerned about the coronavirus”). The lack of consistent responses was a red flag indicating likely failure to sufficiently attend to the survey. However, rating an item phrased in negation terms may place undue demands on language and executive functions. Because these participants successfully passed the attention check and completed the CPT, one might argue that they had paid sufficient attention to the task and should be included.
    The inclusion of these participants increased the sample size of Experiment 2 to 257, but did not change the pattern of results. CPT *A’* did not correlate significantly with latent concerns: *r* = 0.04, *p* = 0.58 with health-related concerns, and *r* = 0.01, *p* = 0.93 with finance-related concerns. Active concerns, however, were more strongly correlated with CPT performance. The correlation of CPT *A’* with general TUT reached significance, Spearman’s *rho* = − 0.190, *p* = 0.002. COVID-specific TUT showed a trend toward a significant correlation with CPT *A’*, *rho* = − 0.110, *p* = 0.08.
5. Other analyses

**Table 4:** Pearson’s correlation coefficient between COVID-19 responses and CPT performance in Experiment 2. Skill index is calculated as No-go accuracy divided by Go RT

Variables	Overall *A’*	Reduction in A’ across blocks	Skill index	No-go (mountain) accuracy	Go (city) RT
*Demographic ratings*					
1. Age	.04	− .04	.02	.12	.23*
2. Education	− .10	.06	− ***.15***	− .09	.07
3. Income	− .004	− .14	− .02	.02	.06
4. Political orientation	− .09	.11	− .09	− .04	.10
5. Health status	.08	− .02	.11	.10	.05
6. Financial wellbeing	− .02	.12	.02	.02	.02
*Concerns related to COVID-19*					
7. Health concern	.01	− .09	− .01	− .02	− .05
8. Financial concern	.01	− .003	.07	.04	− .04
9. Average of all eight items	.09	− .11	.11	.08	− .03
*Behavioral compliance*					
10. Social distancing	− ***.20***	− .002	− ***.21***	− ***.16***	.04
11. Hygienic behavior	.01	.07	− .01	− .01	.01

In the correlation analysis between income and other variables, participants who chose the answer “I prefer not to answer this question” were excluded, leading to *N* = 197. All other analyses had *N* = 204. Only the following correlation reached Bonferroni adjusted alpha: participants with older age showed slower RT in go responses, Pearson’s *r* = .23, *p* = .0007. Boldface italics: *p* < .05 (uncorrected for multiple comparisons)

The scene CPT used in this study was based on previous studies that demonstrated its validity (Rosenberg et al., 2016). As a measure of sustained attention, it should exhibit characteristics comparable to other published CPT variants. One such characteristic is an age effect: older participants tend to have slower response times and lower error rates (particularly commission errors; Carriere et al., 2010; Fortenbaugh et al., 2015). Consistent with these reports, relatively older participants were slower than younger participants, *r* = 0.23, *p* < 0.001 in Experiment 2, and had lower commission error rates, *r* = − 0.21, *p* < 0.008 in Experiment 1.

Finally, to examine whether COVID-19 has lingering cognitive effects, we separated participants who reported to have never experienced COVID-19 symptoms (N = 141 in Experiment 1 and N = 166 in Experiment 2) from participants who did (N = 20 in Experiment 1 and N = 38 in Experiment 2; 14 of the 58 reported to have a confirmed positive COVID test). *A’* did not differ between the two groups, *t*(159) = 1.09, *p* = 0.28 in Experiment 1, *t*(202) = − 0.28, *p* = 0.78 in Experiment 2. Participants reported to have experienced COVID-19 symptoms showed a greater vigilance decrement across blocks only in Experiment 1, *t*(159) = 2.08, *p* = 0.04, but not in Experiment 2, *t*(202) = 0.90, *p* = 0.37. Note that this finding is limited by a lack of clinical verification of the diagnosis. Future research should further examine how a history of infection with COVID-19 affects sustained attention.

## General discussion

Using a continuous performance task, this study measured sustained attention in young adults during the historic COVID-19 pandemic. Experiment 1 was conducted early during the pandemic with participants from 21 predominantly European countries. Participants expressed various levels of concerns about the health risks posed by COVID-19, with some expressing high levels of concern. Yet participants who expressed more health-related or financial concerns about COVID-19 performed at levels comparable to those who were less concerned. Experiment 2 replicated this finding in a different sample—US residents—during a later stage of the pandemic. The second experiment additionally collected data on the self-reported frequency of task-unrelated thoughts (TUT) during an attention task. We found that TUTs were negatively associated with performance on the continuous performance task (CPT). These findings indicate that active concerns may interfere with sustained attention. These concerns manifest as task-unrelated thoughts, a subset of which is COVID-related. However, the frequency of COVID-related TUT was low, suggesting that young adults were largely successful in preventing their latent concerns about COVID-19 from becoming active at least during the task that lasted on the order of 8 minutes, as administered here. Together, these data showed that although COVID-19 induced moderate health and financial concerns, young adults were able to minimize their impact when performing a sustained attention task.

The lack of a significant correlation between pre-task COVID concerns and CPT performance cannot be attributed to the use of an easy or unreliable task. The scene CPT used here was highly demanding. Within just two minutes on the task, accuracy declined significantly. Commission errors—failure to withhold response—reached levels as high as 60%. Not only was the CPT highly demanding, but it also produced large and stable individual differences. Detection sensitivity varied widely from 0.62 to 0.98 (0.50 is chance). This difference across individuals was highly reliable across the two task blocks. In addition, several findings bolster the validity of the scene CPT as a measure of sustained attention. First, similar to other, well-established CPT variants, the scene CPT was sensitive to participants’ age, with older participants producing slower but more accurate responses than younger participants (Carriere et al., 2010; Fortenbaugh et al., 2015). Second, using the gradual-onset version of the scene CPT, a previous study found strong correlations between task performance and everyday attention errors (Rosenberg et al., 2013). In addition, a sustained attention brain network built on the scene CPT successfully predicted ADHD symptoms (Rosenberg et al., 2016). Third, the gradual-onset and clear-view versions of the scene CPT produced highly consistent results. Performance on both tasks correlated with the stop-signal reaction time (Jun & Lee, submitted), a well-established index of attentional inhibition. These properties make the scene CPT an appropriate, and likely sensitive, measure of any disruption on sustained attention.

The weak correlation between scene CPT performance and COVID-related concerns also cannot be attributed to the lack of variability in survey response. The degree of COVID-related concerns spans the entire range of the 7-point rating scale. Items within a given category, such as the two health-related items, produced ratings that were strongly correlated. When we administered both the survey and thought probes in Experiment 2, we found that COVID-related concerns measured in the pre-task survey correlated significantly with the COVID-specific task-unrelated thoughts. This provided additional validation of both measures.

Our study’s finding suggests that current concerns that are latent need not always interfere with demanding attentional tasks. In our study, the correlation between pre-task COVID concerns and CPT *A’* was near zero in the pre-registered, planned analyses. A Bayesian analysis revealed Bayes Factors (BF_01_) ranging from 9.66 to 29.19, suggesting that the lack of a strong correlation was more than 9 times as likely as the presence of a correlation. In addition, the effect size in the correlation was small: variability in COVID-related concerns contributed to no more than 0.5% of the variance in CPT performance. Even if these correlations would become significant with a much larger sample, the size of the effect is too small to carry practical significance.

The inclusion of probes on task-unrelated-thoughts in Experiment 2 helps to elucidate these results. Similar to previous studies, we found that CPT performance was worse for participants with a greater tendency to engage in task-unrelated thinking (McVay & Kane, 2009; Poerio et al., 2013). This finding suggests that active concerns are a source of distraction and can interfere with sustained attention. However, COVID-related concerns measured before the task are a form of latent concerns. Although we found a positive correlation between pre-task COVID concerns and COVID-specific TUTs, the correlation was small in size. In fact, the overall frequency of COVID-specific TUTs was very low. The majority of the participants reported having no active TUTs about COVID-19 during the CPT. This finding suggests that young adults are successful in preventing their pre-task COVID concerns from intruding into the CPT, thus minimizing any impact of those worries on the task.

Our study suggests that current concerns have complex effects on task performance. On one hand, current concerns are a potential source of distraction, reducing attentional resources that are otherwise used on important tasks. On the other hand, these concerns may exist in a latent format. Control mechanisms may exist to suppress or counter the negative impact of current concerns. For example, participants may be able to selectively allocate attention to ongoing tasks, keeping anxiety-provoking thoughts in a dormant form. In fact, engaging in challenging tasks is known to reduce activation in the default-network of the brain (Raichle, 2015), which, in turn, dampens self-referencing or task-unrelated thoughts. Thus, although current concerns can, in principle, interfere with attention to external tasks, their impact may be minimized if proper cognitive control is exerted. This interpretation is consistent with the larger literature on the complex interaction between attention and anxiety (Robinson, Krimsky, et al., 2013; Robinson, Vytal, et al., 2013).

Our finding may be considered inconsistent with resource competition theories. However, the fundamental assumption of these theories—internal states could be a source of distraction—remains valid. Because attention can be directed both internally and externally (Chun et al., 2011), it is likely that current concerns do interact with externally directed attention. What our study shows, however, is that such interactions are more complex than a straightforward prediction based on resource competition. Although it is possible to find positive evidence for resource competition theories (as other studies on mind-wandering have shown), the dynamics governing the interaction between internal states and external attention are likely complex. Under some conditions, and in some individuals, it is possible to optimize performance on an external task even in the face of moderate to severe concerns. Nonetheless, the significant correlation between COVID-related TUT and the pre-task COVID-related concerns suggests that active and latent concerns are closely linked. In fact, the transition between active and latent concerns is likely fluid—the same individual who was able to minimize COVID-related concerns during the scene CPT might experience those concerns during other tasks. There is still much to learn about how participants prevent latent concerns from becoming activated, and how the control mechanisms may interact with the severity of concern, age, and other factors.

Although our study was restricted to young adults, the finding carries significant implications. After all, the young adults tested here represent a wide swath of the population, including college students and recent graduates who frequently perform important sustained attention tasks—driving, studying, and working. Our finding suggests that within this group, the ability to sustain attention remains intact in the face of moderate (but latent) concerns about COVID-19. Our study raises the pressing need to test individuals who are more severely impacted by COVID-19. These include groups who are more concerned about COVID-19, individuals with a confirmed diagnosis of the disease, and those who lost jobs. Longitudinal studies that track changes in mental or physical health, as well as changes in sustained attention, will be highly informative. The current finding may be a silver lining for one specific population—young adults with moderate concerns about COVID-19. They may continue to maintain unaltered standards in sustained attention tasks, such as driving, learning, and working. However, a full understanding of how COVID-19 affects mental health, and in turn, cognitive performance, will require the testing of additional groups and other attention tasks.

## Supplementary Information

**Additional file 1**. COVID-19 survey, demographic information, and additional analyses.

## Data Availability

Pre-registration of the experiments can be found on Open Science Framework (https://osf.io/ay8pq/). The site also contains experimental scripts used for the CPT and documentation of script accuracy. De-identified experimental data in an aggregated format are available from the corresponding author on reasonable request.

## Footnotes

1 The preregistration had a planned sample size of 160 and 200 in Experiments 1 and 2, respectively, after excluding participants who did not meet the inclusion criteria. We stopped data collection after these numbers were reached. However, further analyses found that 5 of the excluded participants met inclusion criteria. These participants were included in the analysis, yielding a final sample size of 161 in Experiment 1 and 204 in Experiment 2.
