# Supplementary material for: Do concerns about COVID-19 impair sustained attention?
Source: Cogn Res Princ Implic. 2021 May 27;6:41. doi: 10.1186/s41235-021-00303-3 (PMC8159070; doi:10.1186/s41235-021-00303-3)
Supplement: Supplementary file 1 — Additional file 1. COVID-19 survey, demographic information, and additional analyses. [file 41235_2021_303_MOESM1_ESM.docx]

**Supplementary Materials**

1. **COVID-19 survey**

- The following questions were in the survey of Experiment 1.

1. Please specify your gender:
   1. Male
   2. Female
   3. Other: ________
2. In which country do you currently reside?
3. In which state do you currently reside? (If you do not reside in the United States, please choose the first option.)
4. Choose one or more races that you consider yourself to be:
   1. White
   2. Black or African American
   3. American Indian or Alaska Native
   4. Asian
   5. Native Hawaiian or Pacific Islander
   6. Other: ________
5. Are you of Hispanic or Latino descent - that is, Mexican, Mexican American, Chicano, Puerto Rican, Cuban, South or Central American or other Spanish culture or origin?
   1. Yes
   2. No
6. What is the highest level of education you completed?
   1. Some grade school
   2. Some high school
   3. High school diploma or GED
   4. Some college or 2-year degree
   5. 4-year college graduate
   6. Some school beyond college
   7. Graduate or professional degree
7. Please indicate your approximated total annual household income before taxes.
   1. Less than $10,000
   2. $10,000 to $19,999
   3. $20,000 to $29,999
   4. $30,000 to $39,999
   5. $40,000 to $49,999
   6. $50,000 to $59,999
   7. $60,000 to $69,999
   8. $70,000 to $79,999
   9. $80,000 to $89,999
   10. $90,000 to $99,999
   11. $100,000 to $149,999
   12. $150,000 or more
   13. I prefer not to answer this question
8. Here is a 7-point scale on which the political views that people might hold are arranged from extremely liberal (left) to extremely conservative (right). Where would you place yourself on this scale?
   1. Extremely Liberal
   2. Liberal
   3. Slightly Liberal
   4. Neutral
   5. Slightly Conservative
   6. Conservative
   7. Extremely Conservative
9. Each statement below is asking what has changed in your infection history since the coronavirus disease pandemic began. Please check Yes (if you were impacted) or No (if you were not impacted).

|  | Yes | No |
| --- | --- | --- |
| 1. Currently have symptoms of this disease but have not been tested. |  |  |
| 1. Tested and currently have this disease. |  |  |
| 1. Had symptoms of this disease but never tested. |  |  |
| 1. Tested positive for this disease but no longer have it. |  |  |
| 1. Got medical treatment due to severe symptoms of this disease. |  |  |

1. Listed below are statements about the impact of coronavirus (COVID-19) on your life. For each one, please indicate how strongly you agree or disagree with each statement (1 = Not true of me at all, 7 = Very true of me). [* In Experiment 2, the scales were labeled as “1 = I am not concerned at all about this possibility” and “7 = I am extremely concerned about this possibility.”]

|  | 1 | 2 | 3 | 4 | 5 | 6 | 7 |
| --- | --- | --- | --- | --- | --- | --- | --- |
| 1. I am worried that I or people I love will get sick from the coronavirus. |  |  |  |  |  |  |  |
| 1. I am worried that the coronavirus will delay the treatment of other illnesses that I or people I love may have. |  |  |  |  |  |  |  |
| 1. I have lost job-related income due to the coronavirus. |  |  |  |  |  |  |  |
| 1. This is a data quality check. Please choose 2. |  |  |  |  |  |  |  |
| 1. I am not concerned about the coronavirus. |  |  |  |  |  |  |  |
| 1. I am concerned about my job security due to the coronavirus. |  |  |  |  |  |  |  |
| 1. I am stressed around people outside of my household because I worry I'll catch the coronavirus. |  |  |  |  |  |  |  |
| 1. I have tried hard to avoid people outside of my household because I don't want to get sick. |  |  |  |  |  |  |  |
| 1. I am worried about the coronavirus. |  |  |  |  |  |  |  |

1. The following questions ask about your experiences with social distancing. Social distancing means keeping space between yourself and other people outside of your home.

|  | None of  the days  (0 days) | A few days (1-2 days) | Most days (3-4 days) | Every day |
| --- | --- | --- | --- | --- |
| 1. In the last 5 days, I have attended social gatherings, outside my home. |  |  |  |  |
| 1. In the last 5 days, I have gone on shopping trips or outings that were "just for fun." |  |  |  |  |
| 1. In the last 5 days, I have visited nursing homes or long-term care facilities (outside of work duties). |  |  |  |  |
| 1. In the last 5 days, I have been in person-to-person contact with someone who is in a risk group (adults age 50+, people with chronic medical conditions) outside of my household. |  |  |  |  |

1. How often are you doing the recommended pandemic hygiene, like washing hands frequently, avoiding touching your face, covering coughs, and avoiding frequently touched surfaces in public places?
   1. All of the time; I am being extra careful
   2. Most of the time; I try my best
   3. Sometimes; I do it if I think of it
   4. Rarely; I don't worry about these things

- Experiment 2’s survey was similar to the one used in Experiment 1, except that the following items were added.

1. How would you rate your general health?
2. Good
3. Fair
4. Poor
5. During the pandemic, how hard has it been for you to pay for the very basic like food, housing, medical care, and heating/cooling? Would you say…
6. Very hard
7. Hard
8. Somewhat hard
9. Not very hard
10. How would you describe the money situation in your household right now?
11. Comfortable with extra
12. Enough but no extra
13. Have to cut back
14. Cannot make ends meet
15. **Demographic information**

*Table S1.*

*Demographic information of participants in the final sample (N=161 in Experiment 1; N=204 in Experiment 2)*

|  | **Experiment 1** | | | **Experiment 2** | |
| --- | --- | --- | --- | --- | --- |
|  | Frequency | % | Frequency | | % |
| ***Gender*** | | | | | |
| Male | 103 | 63.98 | 82 | | 40.20 |
| Female | 58 | 36.02 | 121 | | 59.31 |
| Nonbinary | 0 | 0 | 1 | | 0.49 |
| ***Continent*** | | | | | |
| Europe | 140 | 86.96 | 0 | | 0 |
| South America | 15 | 9.32 | 0 | | 0 |
| North America | 5 | 3.11 | 204 | | 100 |
| South Africa | 1 | 0.62 | 0 | | 0 |
| ***Race and ethnicity (Participants could choose one or more if applicable)*** | | | | | |
| White | 138 | 85.71 | 144 | | 70.59 |
| Hispanic | 32 | 19.88 | 17 | | 8.33 |
| Asian | 6 | 3.73 | 48 | | 23.53 |
| Black or African American | 3 | 1.86 | 15 | | 7.35 |
| American Indian or Alaska Native | 1 | 0.62 | 1 | | 0.49 |
| Native Hawaiian or Pacific Islander | 0 | 0 | 3 | | 1.47 |
| Other | 15 | 9.32 | 8 | | 3.92 |
| ***Highest level of education*** | | | | | |
| Some grade school | 3 | 1.86 | 1 | | 0.49 |
| Some high school | 14 | 8.70 | 3 | | 1.47 |
| High school diploma or GED | 47 | 29.19 | 16 | | 7.84 |
| Some college or 2-year degree | 39 | 24.22 | 80 | | 39.22 |
| 4-year college graduate | 27 | 16.77 | 61 | | 29.90 |
| Some school beyond college | 6 | 3.73 | 7 | | 3.43 |
| Graduate or professional degree | 25 | 15.53 | 36 | | 17.65 |
| ***Political orientation*** | | | | | |
| Extremely liberal | 2 | 1.24 | 23 | | 11.27 |
| Liberal | 51 | 31.68 | 87 | | 42.65 |
| Slightly liberal | 38 | 23.60 | 28 | | 13.73 |
| Neutral | 45 | 27.95 | 40 | | 19.61 |
| Slightly conservative | 20 | 12.42 | 16 | | 7.84 |
| Conservative | 4 | 2.48 | 9 | | 4.41 |
| Extremely conservative | 1 | 0.62 | 1 | | 0.49 |
| ***Annual income before taxes*** | | | | | |
| Less than $10,000 | 43 | 26.71 | 9 | | 4.41 |
| $10,000 to $19,999 | 28 | 17.39 | 6 | | 2.94 |
| $20,000 to $29,999 | 18 | 11.18 | 28 | | 13.73 |
| $30,000 to $39,999 | 19 | 11.80 | 15 | | 7.35 |
| $40,000 to $49,999 | 17 | 10.56 | 15 | | 7.35 |
| $50,000 to $59,999 | 5 | 3.11 | 23 | | 11.27 |
| $60,000 to $69,999 | 4 | 2.48 | 16 | | 7.84 |
| $70,000 to $79,999 | 2 | 1.24 | 18 | | 8.82 |
| $80,000 to $89,999 | 0 | 0 | 11 | | 5.39 |
| $90,000 to $99,999 | 3 | 1.86 | 10 | | 4.90 |
| $100,000 to $149,999 | 1 | 0.62 | 21 | | 10.29 |
| $150,000 or more | 2 | 1.24 | 25 | | 12.25 |
| I prefer not to answer this question | 19 | 11.80 | 7 | | 3.43 |
| ***Difficulty of paying for the basics like food and medicine*** | | | | | |
| Not very hard | - | - | 127 | | 62.25 |
| Somewhat hard | - | - | 52 | | 25.49 |
| Hard | - | - | 17 | | 8.33 |
| Very hard | - | - | 8 | | 3.92 |
| ***Household financial status*** | | | | | |
| Comfortable with extra | - | - | 65 | | 31.86 |
| Enough but no extra | - | - | 87 | | 42.65 |
| Have to cut back | - | - | 43 | | 21.08 |
| Cannot make ends meet | - | - | 9 | | 4.41 |
| ***Overall health status*** | | | | | |
| Good | - | - | 155 | | 75.98 |
| Fair | - | - | 46 | | 22.55 |
| Poor | - | - | 3 | | 1.47 |

1. **Exploratory analysis on participants’ responses to the survey**

To explore how responses to the various survey items related to one another, we conducted pre-registered exploratory correlation analyses between ratings in the demographic questions, ratings in concerns related to COVID-19, and ratings in compliance with social distancing and hygienic recommendations (Experiment 1). In Experiment 2, the exploratory correlation analyses were conducted among the same variables as in Experiment 1, but two more variables (“overall health status” and “financial wellbeing”) were added. The full results of the correlation analyses are presented in Table S2.

Pearson’s correlation that reached the Bonferroni corrected alpha of *p* < .001 included:

1. Age: Older participants had higher education (both experiments) and greater financial concerns (Experiment 1).
2. Education: Higher education positively correlated with higher income (Experiment 2) and greater anxiety around crowds (Experiment 1).
3. Income: People with higher income reported to be in better health (Experiment 2).
4. Political orientation: More conservative individuals were less concerned about COVID-19. This was observed in Experiment 2 (US sample) but not in Experiment 1 (primarily European sample).
5. Overall health and financial well-being (Experiment 2): Participants who reported to be in better health also rated themselves as financially better off. Those doing worse financially had greater concerns about COVID-19’s financial impact.
6. Public health guidance: Participants with greater concerns about COVID-19 were more likely to follow public health guidance regarding hand washing and other hygienic behaviors (both experiments).

*Table S2.*

*Pearson’s correlation coefficients between responses to the COVID-19 survey. Items include demographic questions (1-age, 2-highest level of education, 3-household income, 4-political orientation, 5-overall health status, and 6-financial well-being), COVID-related concerns (7-the average of the two health-related items, 8-the average of the two financial items, 9-the average of the two items assessing anxiety around crowds, and 10-the average of all eight items), compliance with (11) social distancing and (12) hygienic recommendations. *: p < .001 (Bonferroni corrected alpha). Italics: p < .05 (uncorrected for multiple comparisons). Rows in white color indicate the results of Experiment 1; rows in gray color indicate the results of Experiment 2.*

| Variables | 1 | 2 | 3 | 4 | 5 | 6 | 7 | 8 | 9 | 10 | 11 | 12 |
| --- | --- | --- | --- | --- | --- | --- | --- | --- | --- | --- | --- | --- |
| ***Demographic ratings*** | | | | | | | | | | | | |
| 1. Age | – |  |  |  |  |  |  |  |  |  |  |  |
|  | – |  |  |  |  |  |  |  |  |  |  |  |
| 1. Education | **.57*** | – |  |  |  |  |  |  |  |  |  |  |
|  | **.30*** | – |  |  |  |  |  |  |  |  |  |  |
| 1. Income | -.08 | .16 | – |  |  |  |  |  |  |  |  |  |
|  | .05 | **.28*** | – |  |  |  |  |  |  |  |  |  |
| 1. Political orientation | .12 | .02 | -.01 | – |  |  |  |  |  |  |  |  |
|  | ***.17*** | .02 | -.003 | – |  |  |  |  |  |  |  |  |
| 1. Overall health status (†) | – | – | – | – | – |  |  |  |  |  |  |  |
|  | .08 | ***-.22*** | **-.24*** | -.03 | – |  |  |  |  |  |  |  |
| 1. Financial well-being (†) | - | – | – | – | – | – |  |  |  |  |  |  |
|  | ***.18*** | -.12 | -.53 | -.04 | **.26*** | – |  |  |  |  |  |  |
| ***Concerns related to COVID-19*** | | | | | | | | | | | | |
| 1. Health concern | .11 | .06 | -.01 | -.06 | – | – | – |  |  |  |  |  |
|  | -.01 | .09 | .05 | ***-.21*** | ***.16*** | .13 | – |  |  |  |  |  |
| 1. Financial concern | **.26*** | ***.18*** | -.04 | -.03 | – | – | .13 | – |  |  |  |  |
|  | -.05 | -.05 | ***-.18*** | -.05 | ***.17*** | **.42*** | **.28*** | – |  |  |  |  |
| 1. Anxiety around crowds | .06 | **.26*** | -.04 | -.02 | – | – | .**47*** | ***.21*** | – |  |  |  |
|  | .04 | .09 | .07 | **-.27*** | .10 | .12 | **.61*** | ***.21*** | – |  |  |  |
| 1. Average of all eight items | ***.18*** | ***.23*** | -.05 | -.04 | – | – | **.72*** | **.58*** | **.74*** | – |  |  |
|  | -.01 | .04 | -.02 | **-.29*** | ***.18*** | ***.28*** | **.80*** | **.62*** | **.81*** | – |  |  |
| ***Behavioral compliance*** | | | | | | | | | | | | |
| 1. Social distancing (†) | -.02 | -.08 | -.04 | .05 | – | – | -.04 | .02 | ***-.16*** | ***-.16*** | – |  |
|  | .07 | .08 | .12 | ***.19*** | -.07 | -.06 | -.07 | -.01 | ***-.22*** | ***-.17*** | – |  |
| 1. Hygienic behavior (†) | ***-.17*** | ***-.20*** | .***17*** | -.04 | – | – | ***-.23*** | ***-.23*** | ***-.22*** | **-.32*** | .03 | – |
|  | -.06 | -.09 | -.02 | ***.17*** | .13 | -.07 | ***-.31*** | -.11 | **-.48*** | **-.43*** | ***.19*** | – |

*Note: In the correlation analysis between income level and other variables, participants who chose the answer “I prefer not to answer this question” were excluded, leading to N = 142 in Experiment 1 and 197 in Experiment 2. In the other correlation analyses, N was 161 in Experiment 1 and 204 in Experiment 2. Variables with cross (†) require reverse interpretation, in that higher score in the overall health, financial well-being, and behavioral compliance (social distancing and hygienic behavior) represent lower levels of the attribute being evaluated.*

1. **RT adjustment in the scene CPT of Experiment 1**

In the scene CPT, we recorded all responses after the onset of each image. The raw RT ranged from 0 to 800 ms. Following the pre-registered analysis plan of Experiment 1, we assigned each response to either trial N or N+1 based on the following algorithm, adapted from Esterman et al. (2013). First, any response with 100ms < raw RT < 800ms was classified to trial N, based on the assumption that this response was made after the onset of trial N and before the onset of trial N+1. The classified RT for this case was identical to the raw RT. Out of 90,458 trials that received responses, 87,545 trials (96.78%) received responses after the first iteration was completed. Second, 0 ms < raw RT < 50 ms was classified to trial N-1, based on the assumption that this response was made to a previous image. The classified RT for this case was adjusted by adding 800 ms (e.g., a raw RT of 25ms recorded on trial N was adjusted as a classified RT of 825ms made to trial N-1). 1,148 trials (1.27% out of responded trials) received responses after the second iteration was completed. Third, the remaining responses with 50ms < raw RT < 100ms were classified to an adjacent trial that did not yet have a response. If both adjacent trials did not have a response, a response was assigned to an adjacent trial that presented a city image. If both adjacent trials did not have a response and both trials presented scene images in the same category, the response was assigned to the temporally closer trial. 1,765 trials (1.95% out of responded trials) received responses after the third iteration was completed. Finally, if there were multiple presses within a trial, the fastest one was assigned to that trial. This happened on 1,002 trials (1.11%).

Following the pre-registered analysis plan of Experiment 2, we did not apply the RT adjustment in Experiment 2. Instead, the response recorded during a given trial’s image presentation was assigned to that trial, no matter what its RT was. This decision was made based on two factors. First, the adjustment in Experiment 1 affected only a small proportion of trials and did not influence the pattern of the results. Second, the unadjusted RT analysis was simpler, making it easier to adopt in future replication studies.

1. *d’* analysis

To address possible objections to the use of *A’* as a measure of detection sensitivity (Verde et al., 2006), we analyzed *d’* in the scene CPT. In our study, *d’* was strongly correlated with *A’* (Pearson’s *r* = .96 in Experiment 1 and *r* = .95 in Experiment 2). The two measures yielded the same pattern of results.

First, like *A’*, *d’* was strongly correlated between the two CPT blocks, Pearson’s *r* = .64, *p* < .001 in Experiment 1, Pearson’s *r* = .71, *p* < .001 in Experiment 2. In addition, *d’* significantly declined both between blocks, *F*(1, 160) = 28.64, *p* < .001, *η_p_* ^2^ = .15 in Experiment 1, *F*(1, 203) = 57.13, *p* < .001, *η_p_* ^2^ = .22 in Experiment 2, and within a block, *F*(1, 160) = 74.35, *p* < .001, *η_p_* ^2^ = .32 in Experiment 1, *F*(1, 203) = 49.54, *p* < .001, *η_p_* ^2^ < .20 in Experiment 2. In addition, the frequency estimation error did not correlate with *d’*, Pearson’s *r* = -.09, *p* = .25 in Experiment 1, and Pearson’s *r* = -.01, *p* = .94 in Experiment 2.

Second, the severity of COVID-related concerns in the pre-task survey did not correlate with CPT *d’* in either experiments. The correlation between *d’* and health concerns was Pearson’s *r* = -.03, *p* = .74 in Experiment 1, and Pearson’s *r* = .004, *p* = .96 in Experiment 2. The correlation between *d’* and financial concerns was Pearson’s *r* = .10, *p* = .21 in Experiment 1, and Pearson’s *r* = .01, *p* = .85 in Experiment 2.

Third, active concerns, as assessed by the TUT after each block of the CPT in Experiment 2, significantly correlated with *d’*. General TUT correlated negatively with *d’*, Spearman’s *rho* = -.17, *p* = .018. A similar trend was observed between the COVID-specific TUT and CPT *d’*, Spearman’s *rho* = -.12, *p* = .078.


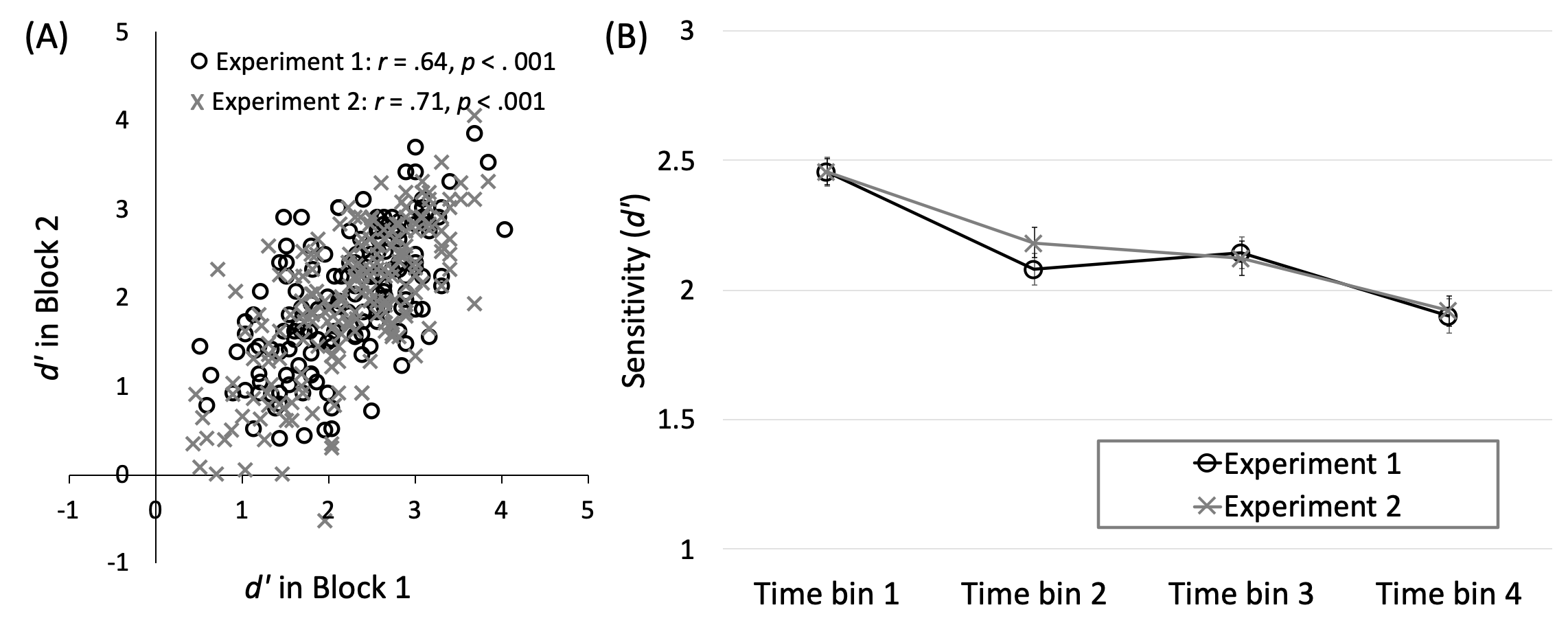


*Figure S1. Scene CPT d’ in Experiments 1 and 2. (A) Scatterplot illustrating the correlation in d’ between block 1 and block 2; (B) Changes in d’ across the four 2-min-long time bins. Error bars show ±1 S.E of the mean. Some error bars may be too small to see.*

1. **RT analysis in the scene CPT**

Figure S2 plots mean RT on trials with correct responses to cities and incorrect responses to mountains. An ANOVA using block (first vs. second) and time bin (first vs. second time bin of each block) as factors showed that there was a significant reduction in RT as time on task increased. In Experiment 1, both the correct RT to cities, *F*(1, 160) = 53.17, *p* < .001, *η_p_* ^2^ = .25, and the incorrect RT to mountains, *F*(1, 159) = 30.57, *p* < .001, *η_p_* ^2^ = .16, became faster in block 2 than block 1^[[1]](#footnote-1)^. Similarly, both the correct RT to cities, *F*(1, 160) = 18.85, *p* < .001, *η_p_* ^2^ = .11, and the incorrect RT to mountains, *F*(1, 159) = 10.79, *p* < .002, *η_p_* ^2^ = .06, became faster in the second than the first time bin. The same pattern of results was observed in Experiment 2.The correct RT to cities became faster in block 2 than in block 1, *F*(1, 203) = 75.70, *p* < .001, *η_p_* ^2^ = .27, and in time bin 2 than in time bin 1, *F*(1, 203) = 71.46, *p* < .001, *η_p_* ^2^ = .26. The incorrect RT to mountains became faster in block 2 than in block 1, *F*(1, 203) = 32.13, *p* < .001, *η_p_* ^2^ = .14, and in time bin 2 than in time bin 1, *F*(1, 203) = 19.49, *p* < .001, *η_p_* ^2^ = .09.

*Figure S2. Time-on-task effect in the mean RT of the scene CPT in Experiment 1 (black lines) and Experiment 2 (gray lines). Error bar shows ±1 SE of the mean across participants.*

1. In Experiment 1, one participant did not make any incorrect responses in the first time bin of block 1. Thus, N equals 160 in this analysis. [↑](#footnote-ref-1)
